# Mycorrhiza‐mediated interference between cover crop and weed in organic winter cereal agroecosystems: The mycorrhizal colonization intensity indicator

**DOI:** 10.1002/ece3.5125

**Published:** 2019-04-30

**Authors:** Alessandra Trinchera, Corrado Ciaccia, Elena Testani, Valentina Baratella, Gabriele Campanelli, Fabrizio Leteo, Stefano Canali

**Affiliations:** ^1^ CREA Research Centre for Agriculture and Environment Council for Agricultural Research and Economics Rome Italy; ^2^ CREA Research Centre for Vegetable and Ornamental Crops Council for Agricultural Research and Economics Monsampolo del Tronto (AP) Italy

**Keywords:** agroecological indicator, AMF, cover crop, rye, SEM, spelt

## Abstract

The mycorrhizal fungi are symbiotic organisms able to provide many benefits to crop production by supplying a set of ecosystem functions. A recent ecological approach based on the ability of the fungi community to influence plant–plant interactions by extraradical mycelium development may be applied to diversified, herbaceous agroecosystems. Our hypothesis is that the introduction of a winter cereal cover crop (CC) as arbuscular mycorrhizal fungi (AMF)–host plant in an organic rotation can boosts the AMF colonization of the other plants, influencing crop–weed interference. In a 4‐years organic rotation, the effect of two winter cereal CC, rye and spelt, on weed density and AMF colonization was evaluated. The AMF extraradical mycelium on CC and weeds roots was observed by scanning electron microscopy analysis. By joining data of plant density and mycorrhization, we built the mycorrhizal colonization intensity of the Agroecosystem indicator (MA%). Both the CC were colonized by soil AMF, being the mycorrhizal colonization intensity (*M*%) affected by environmental conditions. Under CC, the weed density was reduced, due to the increase of the reciprocal competition in favor of CC, which benefited from mycorrhizal colonization and promoted the development of AMF extraradical mycelium. Even though non‐host plants, some weed species showed an increased mycorrhizal colonization in presence of CC respect to the control. Under intense rainfall, the MA% was less sensitive to the CC introduction. On the opposite, under highly competitive conditions, both the CC boosted significantly the mycorrhization of coexistent plants in the agroecosystem. The proposed indicator measured the agroecological service provided by the considered CCs in promoting or inhibiting the overall AMF colonization of the studied agroecosystems, as affected by weed selection and growth: It informs about agroecosystem resilience and may be profitably applied to indicate the extent of the linkage of specific crop traits to agroecosystem services, contributing to further develop the functional biodiversity theory.

## 1 INTRODUCTION

In soil, the mycorrhizal fungi are key functional group, able to support the ecosystem services by activating beneficial symbiotic associations with many plant species (Jeffries, Gianinazzi, Perretto, Tournau, & Barea, 2003). At the same time, the high accessibility of the mycorrhizal fungi to soil resources through external hyphae development allows them to benefit of the photosynthetic carbon produced by the host plants through the fungal symbiosis (Smith & Read, 2008).

Among the mycorrhizas, the arbuscular mycorrhizal fungi (AMF) privilege the root colonization of many herbaceous plant species, such as cereals (Pellegrino, Öpik, Bonaria, & Ercoli, 2015) or vegetables (Baum, El‐Tohamy, & Grudac, 2015), improving plant water and nutrient uptake, modulating competition among organisms (Stampe & Daehler, 2003), regulating allelopathic interactions (Lehman, Taheria, Osborne, Buyerb, & Douds, 2012; Veiga, Jansa, Frossard, & Heijden, 2011), and plant defense (Babikova et al., 2013; Jung, Martinez‐Medina, Lopez‐Raez, & Pozo, 2012).

**Figure 1:**
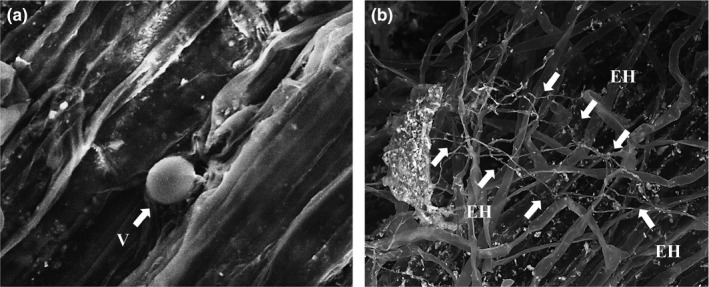
Arbuscular mycorrhizal fungi (AMF) vesicle (a) in a *Triticum durum* root cell (scanning electron microscopy‐back‐scattered electrons [SEM‐BSE], Mag = 4.0 KX) and AMF extraradical hyphal (EH) network developed on wheat roots (b) (SEM‐BSE, Mag = 1.0 KX)

Referring to natural systems, a “social” role was attributed to the mycorrhizal mycelium network in facilitating and influencing plant organisms interactions, by affecting seedling establishment, altering plant–plant interactions, supplying and recycling nutrients (van der Heijden & Horton, 2009; Simard & Durall, 2004): This network development is mainly associated with the ectomycorrhizal fungi colonization in natural ecosystems (Simard & Durall, 2004). The AMF, as endomycorrhizal fungi, predominantly colonize the host plant roots at first, by forming internal structures such as arbuscules, vesicles (Figure 1a), intra‐ and interhyphae, and then growing out to develop a complex, ramified extraradical hyphal network into the surrounding soil (Figure 1b), which can reach up to 30 m of fungal hyphae per gram of soil (Dai et al., 2013; Gianinazzi et al., 2010).

The influence of this AMF extraradical mycelia on the underground biogeochemical cycling and the composition of plant communities, as well as its role as ecological service provider, are widely recognized, particularly in organic cropping systems: root morphology modification, increasing mineral nutrient and water uptake, buffering effect against abiotic stress, protecting against root pathogens (Gianinazzi et al., 2010; Gosling, Hodge, Goodlass, & Bending, 2006; Leake et al., 2004).

The plant host–fungi contact is a crucial moment between the partners in activating the AMF–plant symbiosis, being mediated by the ability of the host plant to exude the “branching factor,” such as the strigolactones, molecules also responsible of the seed germination of parasitic weeds (Akiyama & Hayashi, 2006; García‐Garrido, Lendzemo, Castellanos‐Morales, Steinkellner, & Vierheilig, 2009; Martin et al., 2001). On the contrary, the AMF non‐host plants theory is mainly based on the ability to exude infection‐inhibiting factors by the non‐host species, such as the glucosinolate compounds produced by the *Brassicaceae* (Afzal, Bajwa, & Javaid, 2000; Bajwa, Javaid, Tasneem, & Nasim, 1996; Jafariehyazdi & Javidfar, 2011; Javaid, 2008; Javaid & Riaz, 2008).

Recently, by exploring the function of root exudates in modulating the development of plants, an interesting theory was formulated on the role of mycorrhizal network in transferring plant allelochemicals. These compounds influence the germination, growth, survival, and reproduction among plants (Putnam, 1988; Singh, Batish, & Kohli, 2010) from donors to target plants through extension of a bioactive zone, defined as “fungal fast lane” (Allen, 2007; Barto et al., 2011; Giovannetti, Sbrana, Avio, & Strani, 2004). This privileged transfer, promoted by water diffusion through mycorrhizal hyphae surface, or by the fungal hyphae through internal cytoplasmic flow, leads to accumulation of allelochemicals within the crop–weed rhizosphere at levels that could not be reached by the mere diffusion through the bulk soil (Achatz, Morris, Müller, Hilker, & Rillig, 2014). At ecological level, this fascinating theory gives a key role to AMF extraradical mycelium, as a means of potential long‐distance communication among coexistent plants for reaching space, water, and nutrient resources, influencing their reciprocal inhibition or growth (Barto et al., 2011).

This eco‐social approach could be profitably applied to diversified, organic cropping systems, where agroecological service crops (i.e., living mulch, cover crops [CC]) are used for managing weeds by exploiting allelopathic and competitive interactions among coexisting plant species (Flash, 1990). The mechanisms of interaction underlying the ability of winter cereal CCs to contain weed were deeply investigated: some cereals, such as rice (*Oryza sativa* L.), barley (*Hordeum vulgaris* L.), spelt (*Triticum dicoccum *L.), and rye (*Secale cereale* L.) can reduce weed growth through competition and allelopathic interactions (Barnes, Putnam, Burke, & Aasen, 1987; Chung et al., 2002; Ciaccia et al., 2015; Creamer, Bennett, Stinner, Cardina, & Regnier, 1996; Jung, Kim, Ahn, Hahn, & Chung, 2004; Petersen, Belz, Walker, & Hurle, 2001). It was also found that the introduction of minimum tillage and CCs in conservative agroecosystems increases the diversity and the abundance of AMF in soil (van der Heijden, Boller, Weimken, & Sanders, 1998; Jordan, Zhang, & Huerd, 2000). Other recent in field studies demonstrated that, under organic management, the intercropped CC increased the mycorrhizal colonization of the cash crop, because of positive rhizosphere interactions among diversified plants in reduced volume of soil (Trinchera et al., 2016). In addition, the ability of the rye to increase the AMF colonization in herbaceous system was already observed (Lehman et al., 2012; White & Wei, 2009). However, the relationship between AMF colonization and interference phenomena is not completely clarified and further studies in field are needed to evaluate the effect of CCs introduction on mycorrhizal colonization in conservative agroecosystems (Javaid, 2008; Javaid & Riaz, 2008; Jung et al., 2012; Khanh, Chung, Xuan, & Tawata, 2005; Lehman et al., 2012; Veiga et al., 2011).

On the assumption that soil–fungi—plant relationship is boosted by the organic management, we hypothesized that, where rye and spelt are introduced as winter cereal CCs in an organic rotation, the overall mycorrhizal colonization of the agroecosystem increases by extraradical mycelium development, thus affecting weed emergence, density, and species selection. To verify our hypothesis, a new indicator was developed, linearly correlated to the density of coexistent plant species and their mycorrhization (Duelli & Obrist, 2003), to assess the ecological service provided by the CC on the belowground functional biodiversity under organic management.

## 2 MATERIALS AND METHODS

### 2.1 Site description and experimental design

A 2‐year field experiment was carried out in switching fields at the MOnsampolo VEgetables organic Long‐Term field Experiment (MOVELTE), at the CREA Research Centre for Vegetable and Ornamental Crops (Council for Agricultural Research and Economics), located in Monsampolo del Tronto (AP), coastal area of Central Italy (42°53′N, 13°48′E) characterized by a typical thermo‐Mediterranean climate.

The soil at the field trial was Typic Calcixerepts fine‐loamy, mixed thermic one (USDA, 1996). The min and max temperatures range between 0°C in winter and 40°C in summer (*T*_av_ = 9.9°C in November 2013–April 2014 and 9.5°C in November 2014–April 2015). The rainfall distribution throughout the year is uneven, being the autumn season most predominant, with a total rainfall of 786.4 mm in 2014 and 483.7 mm in 2015. From November 2013 to April 2014, corresponding to the first winter cereal cropping cycle, rainfall was not equally distributed (360 mm only on November 2013) and globally higher (800 mm) respect to the second cycle (485 mm from November 2014 to April 2015, with 200 mm on February).

In a 4‐year organic rotation, our 2 years experiment consisted in a randomized block design (RBD), with three adjacent blocks (linear gradient). Two autumn–winter cereals, spelt (*T. dicoccum* L.) and rye (*S. cereale* L.) were used as CCs, compared to a control treatment without CCs (CNT). Each treatment (CNT, spelt and rye) was randomly replicated once in the three blocks (in total: nine plots). The same experimental design was repeated in 2014 and 2015 in two adjacent fields within the MOVELTE site.

Cover crop was sown at the same rate of 250 kg/ha in both the first (2013) and second (2014) year. During the CC cycles, no weeding was carried out in plot treatments. In both the years of the trial, CCs were terminated in May, before the next vegetable crop of the rotation (Campanelli & Canali, 2012).

### 2.2 Weeds and cover crops density

The five most abundant and representative weed species among the treatments were considered as reference weeds of the studied agroecosystems: *Rumex crispus* L., *Stellaria media* L., *Veronica persica *L., *Polygonum aviculare* L., and *Anagallis arvensis* L.

In both years, 175 days after sowing (DAS: end of April 2014–2015), corresponding to rye full flowering and spelt boot, density of spelt and rye, as the number of plants (pp) per unit of surface (*D*_CC_, pp × m^−2^), the density of total weed species (*D*_WEED‐TOT, _pp × m^−2^), and the specific weed_i _density (*D*_WEED_ *_i_*, pp × m^−2^) were recorded within a 0.25 × 0.25 m^2^ area with three replicates per plots, corresponding to a total of nine records per treatment.

In addition, the weed relative abundance was calculated as the ratio between the number of considered weeds and the total weeds, per surface unit (RA%).

### 2.3 Mycorrhizal colonization intensity (*M*%)

To quantify the root mycorrhizal colonization intensity (*M*%) of each plant species, in both 2014 and 2015, at 175 DAS, the root apparatus of CC and weeds was sampled from field by using stainless steel cylinders of 6 cm diameter and 20 cm length (Trinchera et al., 2016). Per each CC and weed species, three root subsamples per plot were collected, then pooled to obtain *n*. 1 root sample × *n*. 3 treatments × *n*. 3 blocks (nine records/plant species). Roots were immediately separated from the soil by washing in distilled water in a sieve of 0.5 mm mesh and then ordinated into first‐, second‐, and third‐order lateral roots for further analyses.

At random, from each pooled root sample of CC and weed, a total of 10 × 1 cm root pieces per plant (third‐order lateral roots, diameter <2 mm) were cut from 5 to 15 mm from the root tip by a razor blade. The root fragments were stained using a solution of 0.05% w/v methyl blue in lacto‐glycerol (1:1:1 lactic acid, glycerol, and water) for 1 min and destained by distilled water for 1 min more (Grace & Stribely, 1991). Then, the root fragments were placed on grinded slides, mounted in a drop of glycerol, and observed under a light microscope (Nikon E100). The mycorrhizal colonization intensity of CC (*M*_CC_ *_i_*%) and weed (*M*_WEED_ *_i_*%) roots was assessed by applying the method of Trouvelot, Kouch, and Gianinazzi‐Pearson (1986), based on the observation of the root fragments occupied by AMF structures. For each “*i*” plant species and treatment, the total number of observed fragments was three plants × three blocks × 10 = 90. *M_i_*% was calculated attributing to each root fragment increasing scores from 0 to 5:

0 = no AMF structures within the root segment; 1 = structures occupy <1% of the root segment; 2 = structures occupy <10% of the root segment; 3 = structures occupy <50% of the root segment; 4 = structures occupy more than 50% of the root segment; 5 = structures occupy more than 90% of the root segment.

The *M_i_* (as %) for each “*i*” plant species was calculated as follows:

$$M_{i}=\left(95n5+70n4+30n3+5n2+n1\right)/\text{total}\;\text{number}\;\text{of}\;\text{observed}\;\text{fragments}$$

where *n*5 is the number of fragments rated 5, *n*4 is the number of fragments rated 4, and so on.

Quantitative data on the CC and weeds AMF arbuscular richness (here not reported) were collected to verify if the observed external mycelium on roots was due to AMF colonization, in particular in those weeds usually recognized as non‐host plants (as in *R. crispus,* *S. media* and *P. aviculare*).

### 2.4 Scanning electron microscopy (SEM) of extraradical hyphae on mycorrhizal roots

To obtain a visual evidence of the development of AMF extraradical hyphae (ext‐hyp) on coexistent plants in the considered agroecosystems, a comparison among root fragments of the five weed species under both the CC and in the unweeded CNT was carried out; in addition, the extraradical mycelium on rye and spelt root cortex was observed.

For each mycorrhized plant, 3 × 1 cm root pieces (third‐order fine lateral roots, diameter <2 mm) were observed by scanning electron microscope (Microscope Zeiss—EVO MA10) under variable pressure equipped with a LaB_6_ electron sources by using the back‐scattered electrons detector (SEM‐BSE). The applied variable pressure mode (at 20–25 kV EHT/10 Pa chamber pressure) prevented surface damages of such biological and non‐conductive samples, giving a high resolution images without any prior sample preparation. SEM was also implemented by the Beam Sleeve technology, able to extend the vacuum column to 2 mM of the specimen for improving contrast and analytical accuracy. The use of the BSE detector was coupled with a LaB_6_ electrons source, which guaranteed a very high brilliance, through optimizing the performances of back‐scattered electron microscopy. The SEM analysis at 700X and 1.2KX magnification allowed to observe the ultrastructural root surface and thus in particular the extraradical hyphae without any pretreatment of the root fragments, avoiding mechanical stress on root surface and guaranteeing the conservation of the original morphology of the hyphal apparatus. Images were kept on weed roots sampled in April 2014, except for *V. persica *under rye and spelt, which was sampled in April 2015.

### 2.5 Mycorrhizal colonization intensity of the agroecosystem (MA%)

The MA% indicator is defined as the mycorrhizal colonization intensity of the agroecosystem. When compared to the treatment without CCs (here, the CNT), it measures the agroecological service supplied by the CC introduction, in terms of increased mycorrhization of coexistent plants in the agroecosystem, thus affecting the plant–plant interference. It was obtained by weighting the contribution of each plant species, whose density derives from the field interference, to its mycorrhization. In‐field and in‐lab data were aggregated, namely the total density of all plant species (CC and weeds), the specific density of the five most representative weed species, and the root mycorrhizal colonization intensity of CC and weeds, as result of the plant–plant interspecies rhizosphere interaction within each designed agroecosystems.

The contribution of each plant species (CC or weed) to the mycorrhization of the agroecosystem was calculated as (*M_i_* × *D_i_*), assuming 100% the sum of contributions of considered plant species. The *M_i_* × *D_i_* was used as an endpoint: The higher it was for the “*i*” plant species, the higher was the “*i*” plant contribution to the AMF colonization of the agroecosystem.

As a quantitative aggregate function, the MA, expressed as percentage, weights the contribution of each plant species to the mycorrhizal colonization of the whole agroecosystem. This agroecological indicator is referred to the surface soil unit (i.e., per m^2^), calculated in accordance to the below‐reported function:

$$\text{MA}\left(\%\right)=\frac{\left[\sum \left(M_{\text{WEED}i}\times D_{\text{WEED}i}\right)\right]+\left[\sum \left(M_{\text{CC}i}\times D_{\text{CC}i}\right)\right]}{D_{\text{WEED - TOT}}+D_{\text{CC}i}}$$

where: *M*_WEED_ *_i_* is the mycorrhizal colonization intensity of the “*i*” weed, in %; *D*_WEED_ *_i_* is the specific “*i*” weed density (*N*_plant_/m^2^); *M*_CC_ *_i_* is the mycorrhizal colonization intensity of the “*i*” CC, in %; *D*_CC_ *_i_* is the density of the “*i*” CC (*N*_plant_/m^2^); *D*_WEED‐TOT_ is the total weed density (*N*_plant_/m^2^).

Evidently, the higher is the number of considered plant species, the more the MA indicator can accurately describe the mycorrhization of the agroecosystem.

### 2.6 Statistical analysis

A preliminary analysis on *D*_CC_, *D*_WEED‐TOT,_ *D*_WEED_ *_i_*_,_ *M*_CC_ *_i_*%, *M*_WEED_ *_i_*%, and MA% drove into separate ANOVAs for 2014 and 2015. Thus, to match the RBD requirements, all tested parameters were statistically analyzed by ANOVA, considering the block (B) as random factor and the treatment (CC) as fixed factor. Mean comparison was carried out according to post hoc Tukey's HSD test using SPSS (IBM Corp., Armonk, NY, USA).

## 3 RESULTS

### 3.1 Weeds and cover crops sampling and density

In both the years, the *D*_CC_ of rye and spelt showed a comparable ability in covering the soil surface, justifying their use as CCs. Both the CC were able to contain weeds: In 2014 and 2015, the *D*_WEED‐TOT_ decreased significantly under rye and spelt respect to the unweeded CNT, the last one giving the highest *D*_WEED‐TOT_ values (Table 1).

**Table 1:** Density of cover crop (*D*_CC_, pp × m^−2^); density of total weed species (*D*_WEED‐TOT_, pp × m^−2^); and relative weed abundance (RA%) at 175 days after sowing (2014–2015)

	*D*_CC _(pp × m^−2^)	*D*_WEED‐TOT _(pp × m^−2^)	RA% (Σ_WEED_ *_i_*/_WEED‐TOT_)*100
2014			
CNT	–	227 ± 83^a^	86 ± 11^a^
Spelt	56 ± 1	83 ± 19^b^	87 ± 9^a^
Rye	60 ± 9	75 ± 50^b^	68 ± 5^b^
CC‐effect (Sig.)	NS	****	*
Block‐effect (Sig.)	NS	NS	NS
2015			
CNT	–	174 ± 43^a^	39 ± 9
Spelt	66 ± 17	100 ± 86^b^	52 ± 13
Rye	80 ± 23	50 ± 26^b^	39 ± 11
CC‐effect (Sig.)	NS	**	NS
Block‐effect (Sig.)	NS	NS	NS

Notes

Different letters represent significant differences (Tukey's HSD test for means comparison).

CC: cover crop.

Levels of statistical significance are

* *p* < 0.05,

** *p* < 0.01, and

*** *p* < 0.001 (ANOVA).

In 2014, the RA% under rye was significantly lower respect to that in CNT and under spelt, while in 2015, no significant differences among the treatments were recorded (Table 1).

Although at different order of magnitude, in 2014 and 2015 the specific *D*_WEED_ *_i_*, calculated for each weed species, was affected by the CC (Table 2). In 2014, the rye and the spelt significantly reduced the growth of *P. aviculare*, *V. persica*, *A. arvensis,* and *R. crispus*, being their specific densities the highest ones in the CNT, while *S. media* was not been affected by both the CC. In 2015, *V. persica *and *A. arvensis *were the more representative plant species in all the considered agroecosystems. The *D*_WEED_ *_i_* of *P. aviculare* and *A. arvensis *were significantly reduced under both the spelt and the rye treatments.

**Table 2:** Specific weed density (*D*_WEED_ *_i_*, pp × m^−2^) of considered weed species at 175 days after sowing (2014–2015)

*D*_WEED_ *_i_* (pp × m^−2^)					
	*Polygonum aviculare*	*Stellaria media*	*Veronica persica*	*Anagallis arvensis*	*Rumex crispus*
2014					
CNT	59 ± 12^a^	35 ± 13	11 ± 6^a^	70 ± 33^a^	19 ± 10^a^
Spelt	5 ± 9^b^	40 ± 14	0^b^	24 ± 8^ab^	3 ± 1^b^
Rye	16 ± 16^b^	19 ± 16	0^b^	11 ± 4^b^	4 ± 2^b^
CC‐effect (Sig.)	***	NS	***	*	**
Block‐effect (Sig.)	NS	NS	NS	NS	NS
2015					
CNT	2 ± 1^a^	1 ± 1	16 ± 5	48 ± 16^a^	2 ± 2
Spelt	0^b^	4 ± 4	23 ± 21	25 ± 8^ab^	0
Rye	0^b^	1 ± 1	7 ± 8	12 ± 8^b^	0
CC‐effect (Sig.)	*	NS	NS	*	NS
Block‐effect (Sig.)	NS	NS	NS	NS	NS

Notes

Different letters represent significant differences (Tukey HSD test for mean comparison).

CC: cover crop.

Levels of statistical significance are

* *p* < 0.05,

** *p* < 0.01, and

*** *p* < 0.001 (ANOVA).

### 3.2 Mycorrhizal colonization intensity of CC and weed (*M*%)

**Figure 2:**
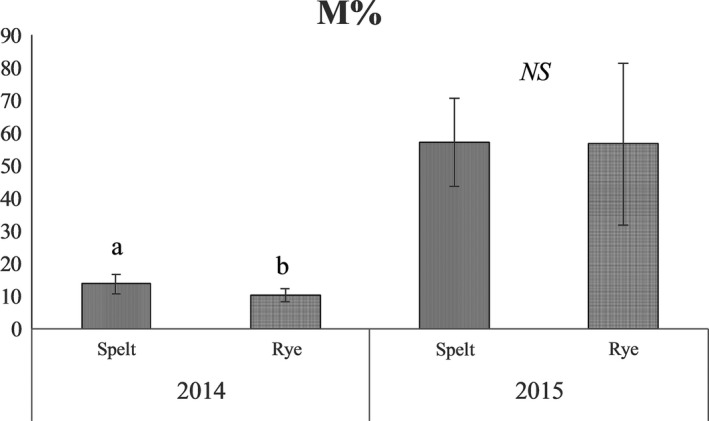
Mycorrhizal colonization intensity of spelt and rye (*M*%) recorded in 2014 and 2015

In 2014, the *M*% of the spelt was significantly higher than that of the rye, while in 2015 no significant differences were recorded between the CC (Figure 2).

**Figure 3:**
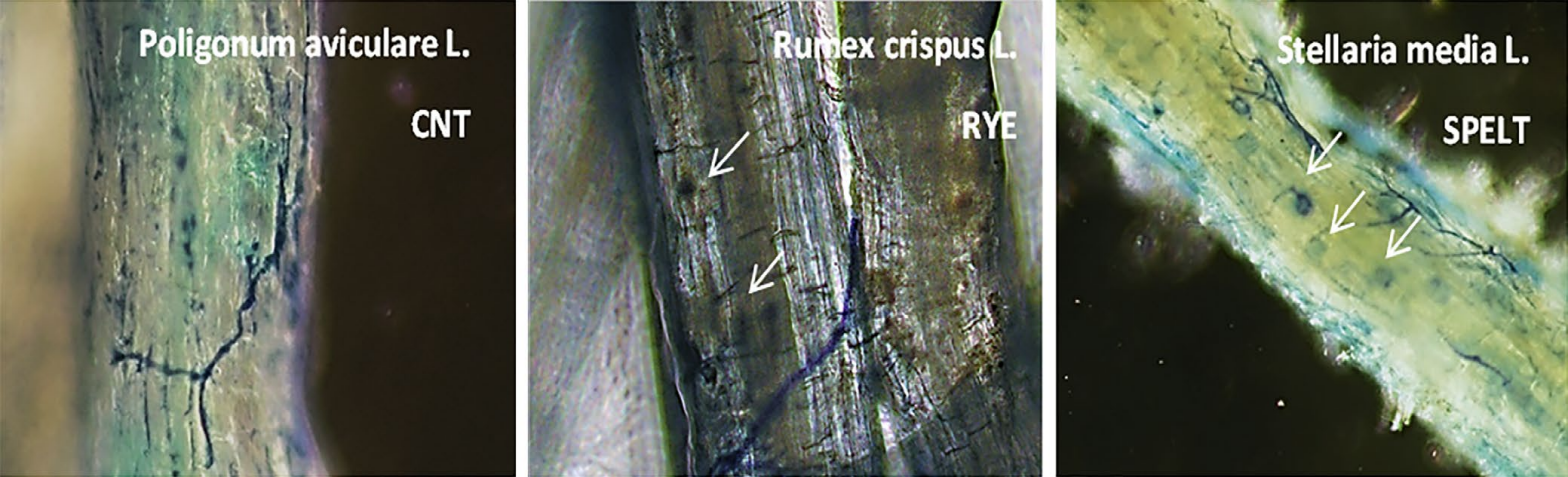
Images of arbuscular mycorrhizal fungi (AMF) colonization of fine lateral roots fragments of *Polygonum aviculare, Rumex crispus, *and *Stellaria media* roots, observed by optical microscope (Mag.:40×). White arrows indicate AMF vesicles and interhyphae

The *M*_weed_% was considered only when intraradical structures, such as interhyphae, coils, and vesicles, were recognized, since the *P. aviculare*, the *R. crispus,* and the *S. media* are known as non‐host endomycorrhizal plant species: These structures were rarely observed in *P. aviculare*, while they were found more frequently in *S. media* and *R. crispus* roots (Figure 3).

In both the years, the *M_i_*% was different among considered weed species, being significantly affected by the CC (Table 3).

**Table 3:** Mycorrhizal colonization intensity (*M*%) of considered weed species at 175 days after sowing (2014–2015)

*M*%					
	*Polygonum aviculare*	*Stellaria media*	*Veronica persica*	*Anagallis arvensis*	*Rumex crispus*
2014					
CNT	2.0 ± 0.8^b^	2.3 ± 1.2^b^	2.1 ± 1.6	21.7 ± 7.4^a^	12.0 ± 7.4^b^
Spelt	13.3 ± 7.2^a^	20.3 ± 9.4^a^	–	2.3 ± 2.2^b^	30.0 ± 3.1^a^
Rye	0.7 ± 0.6^b^	11.7 ± 7.3^ab^	–	3.7 ± 3.0^b^	13.3 ± 5.7^b^
CC‐effect (Sig.)	***	**	–	***	**
Block‐effect (Sig.)	NS	NS	–	NS	NS
2015					
CNT	1.0 ± 0.9	15.1 ± 5.3^ab^	2.4 ± 1.7^a^	56.7 ± 6.8^b^	13.3 ± 10.9
Spelt	–	21.7 ± 6.8^a^	3.3 ± 2.3^a^	78.3 ± 7.0^a^	–
Rye	–	1.0 ± 0.5^b^	0.7 ± 0.6^b^	86.7 ± 14.7^a^	–
CC‐effect (Sig.)	–	**	*	*	–
Block‐effect (Sig.)	–	NS	NS	NS	–

Notes

Different letters represent significant differences (Tukey HSD test for mean comparison).

CC: cover crop.

Levels of statistical significance are

* *p* < 0.05,

** *p* < 0.01, and

*** *p* < 0.001 (ANOVA).

In 2014, the *M*% of *R. crispus *and *P. aviculare *was the highest under spelt and the lowest under rye and in the CNT, while *M*% of *S. media *increased significantly in presence of both the CC. On the opposite, the *M*% of *A. arvensis* was the highest in the CNT respect to those under spelt and rye (Table 3).

In 2015, the *M*% of *A. arvensis*, the most prevalent weed species in all the treatments, was increased under rye and spelt respect to that of the CNT. The *S. media* showed the highest *M*% under spelt, while both *S. media* and *V. persica* gave a decreased *M*% under rye (Table 3).

### 3.3 SEM analysis of extraradical hyphae on mycorrhizal roots

**Figure 4:**
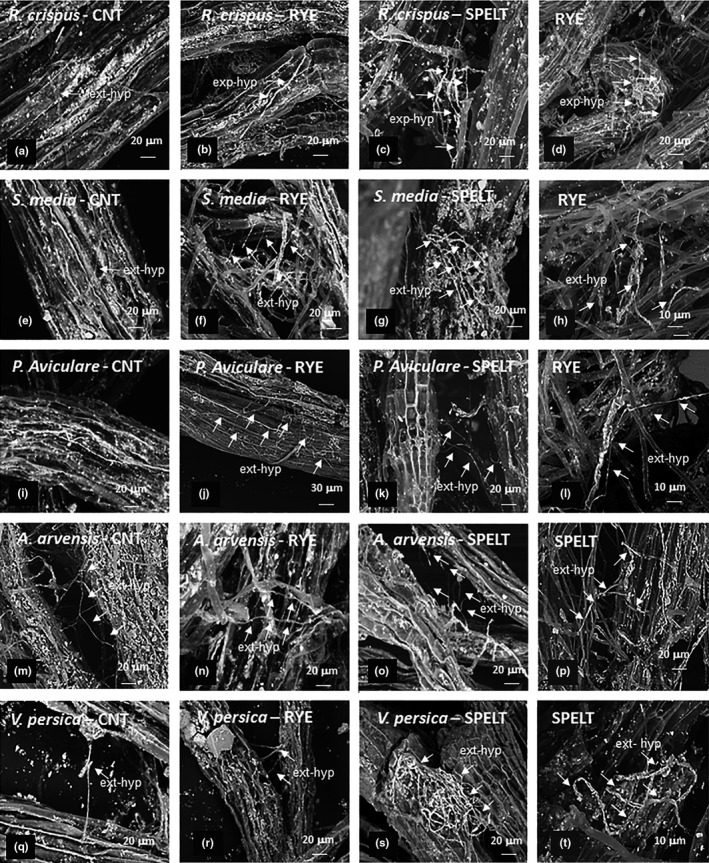
Scanning electron microscopy–back‐scattered electrons (Mag = 700×; s and u: Mag = 1.KX) images of arbuscular mycorrhizal fungi extraradical hyphae (ext‐hyp) on fine lateral roots fragments of: *Rumex crispus, Stellaria media, Polygonum aviculare, Anagallis arvensis, Veronica persica* in CNT (a–e–i–m–q), under rye (b–f–j–n–r) and under spelt (c–g–k–o–s), rye (d–h–l) and spelt (p–t)

The SEM–BSE images of AMF extraradical hyphae of both the CC and the weed (*R. crispus, S. media, P. aviculare, A. arvensis, V. persica*) roots under CNT, rye and spelt, are reported in Figure 4.

Even if commonly recognized as mycorrhizal non‐host plants, *R. crispus *and *P. aviculare* showed limited external mycelium development on root surface in presence of the CCs, particularly under spelt (Figure 4c–k), being absent in the CNT (Figure 4a–i). An increase of extraradical hyphal colonization was also recognizable in *S. media* and *V. persica* under both the CCs (Figure 4f–g–r–s), while in *A. arvensis,* it was well developed in the CNT (Figure 4m) and under CC (Figure 4n–o). Both the CC instead showed an abundant external mycelium on root cortex, together with a proliferation of many root hairs (Figure 4d–t). Some extraradical hyphae connecting adjacent roots in *A. Arvensis* (Figure 4m–o), *V. persica* (Figure 4q–r), *P. aviculare* (Figure 4k), rye (Figure 4l), and spelt (Figure 4p) were observed.

### 3.4 Mycorrhizal colonization intensity of the agroecosystem (MA%)

**Figure 5:**
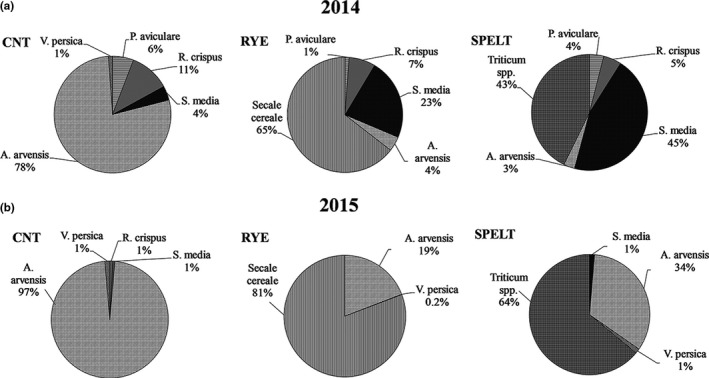
Contribution of cover crop (CC) and weed, calculated as *M_i_* × *D_i_* in 2014 (a) and 2015 (b)

Each *M_i_* × *D_i_* factors in the MA% formula corresponded the contribution of each plant species to the mycorrhization of the considered agroecosystems: Both in 2014 and in 2015, the *A. arvensis* and the *R. crispus *were the weed species which strongly contributed to the mycorrhization of the CNT system (Figure 5a–b).

Going to the cover cropped systems, the contribution of the rye and the spelt to the mycorrhization in field was relevant in both the years. Anyway, under rye only the *A. arvensis *was able to compete and successfully grow, thus increasing the agroecosystem mycorrhization. Under spelt, in 2014 the *S. media* (Figure 5a) and in 2015 the *A. arvensis* effectively contributed, due to the high number of these plants per unit of surface (Figure 5b).

The MA values in cover cropped systems and the CNT, calculated in 2014 and 2015 experiments, are reported in Table 4. The MA% values in 2014 and 2015 strongly differed in terms of magnitude, being in the second year about three times higher than those found in the first one. In 2014, even if a significant CC‐effect was recorded on MA% (*p* = 0.043), the observed significant block‐effect testified the relevance of the variability of mycorrhizal colonization across the field in that year (*p* = 0.008). In 2015, it was significantly affected by CC, increasing +270% under rye and +120% under spelt respect to the CNT.

**Table 4:** Mycorrhizal colonization of the agroecosystem (MA%) in 2014–2015

2014	MA%	2015	MA%
CNT	8.6^ab^	CNT	15.5^b^
Spelt	12.4^a^	Spelt	41.3^a^
Rye	7.0^b^	Rye	50.0^a^
CC‐effect (CC)	*	CC‐effect	*
Block‐effect (B)	**	Block‐effect (B)	NS

Notes

Different letters represent significant differences (Tukey HSD test for mean comparison).

CC: cover crop.

Levels of statistical significance are

* *p* < 0.05,

** *p* < 0.01, and

*** *p* < 0.001 (ANOVA).

## 4 DISCUSSION

Mycorrhizal symbiosis is already recognized as an effective strategy played by AMF plants for overcoming numerous biological (soil microorganisms and fungi biodiversity, allelopathic interaction, etc.) and environmental factors (climatic conditions, competition for light, water, and nutrients, etc.), variable in time and space (Afzal et al., 2000; Baum et al., 2015; Gosling et al., 2006; Leake et al., 2004). In our experimental cover cropped agroecosystem, we verified that this strategy was profitably used by both CC and weeds to obtain a mutual ecological advantage, especially in unfavorable environmental conditions.

Given the effect of CC on plant density and mycorrhization data, the yearly temperatures and precipitations had a key role in modulating CC–weed interference. In 2014, under the sudden and heavy rainfall and in absence of CC (unweeded CNT), weeds had to sustain a reduced competition for water and nutrient, while in 2015 the lowest average rainfalls, with the most abundant precipitation recorded in February (just before the plant sampling date) favored the selection of *V. persica *and the *A. arvensis* (Craine & Dybzinski, 2013). It was noticed that, in both the years, the highest AMF colonization of the *A. arvensis *was positively correlated to its highest density in field: this suggests that *A. arvensis* has been benefited respect to the other weed species by the mycorrhizal colonization, a profitable strategy for optimizing water and nutrient uptake under competition (Allen, Swenson, Querejeta, Egerton‐Warburton, & Treseder, 2003; Marschner & Dell, 1994).

Rye has been able to reduce weeds by homogeneously covering the soil, as confirmed by the highest *D*_CC_, probably exploiting also its recognized allelopathic properties (Barnes et al., 1987; Belz, 2007; Ciaccia et al., 2015; De Albuquerque et al., 2010). However, the lowest *D*_WEED‐TOT_ and RA% suggest that the rye was strongly active, but not selective in containing weed (Cheng & Cheng, 2015; Tabaglio, Marocco, & Schulz, 2013). By comparing each specific weed density to the corresponding mycorrhization intensity, the relationship between weed selection and mycorrhization emerged again under rye: in 2014 the *S. media,* and in 2015 the* A. arvensis,* were the most abundant weed species (25% of the total), with a corresponding increase of mycorrhization of +9.4% in *S. media* and +30% in *A. arvensis *respect to those recorded in the CNT. As a matter of fact, the *Anagallis *genus is characterized by strong allelopathic potential, particularly on gramineous plants, such as millet or wheat: this property, together with the highest weed mycorrhization recorded in field, explain its predominance in such competitive agroecosystem (Rebaz, Shaukat, & Siddiqui, 2001). The other species (i.e., *S. media, P. aviculare *and *R. crispus*), which are generally considered as non‐host endomycorrhizal plants (Ronikier & Mleczko, 2006), showed in our systems intraradical structures usually formed by AMF, such as inter‐radical hyphae, coils and vesicles, rarely in *P. aviculare, *sometimes in *R. crispus *and more frequently in *S. media*. The near absence of *R. crispus* in the rye treatment, regardless of environmental conditions, suggests that its lacking mycorrhization made it sensitive to the allelochemicals exuded by rye (La Hovary et al., 2016). A previous in vitro test on *R. crispus* seeds showed their high sensitiveness to the allelopathic activity of rye, which was not only able to reduce its rootlet elongation, but also to inhibit root fungi colonization at emergence (Trinchera, Testani, Ciaccia, Tittarelli, & Canali, 2015).

The spelt treatment consistently contained weeds, due to its ability to advantageously compete for water, nutrient, and soil niches under rainy conditions (Gross et al., 2010). Given the highest RA% recorded under spelt, particularly in 2014, it was the more selective CC in inhibiting weed growth: Actually, the net prevalence of some weed species found in both the years (*S. media* and *A. arvensis* in 2014, while *V. persica* and *A. arvensis *in 2015) and the quite absence of the others (*P. aviculare* and *R. crispus*) allowed to infer that the spelt promoted specific spelt–weed association for optimizing the resource uptake (Allen & Allen, 1984). In both the years, the highest spelt *M*% corresponded to the higher mycorrhization of the coexistent weeds, compared to that recorded in the CNT: this is another evidence of the synergic, and not antagonistic, CC/weed interaction, mediated by root mycorrhization.

The increase of *M*_WEED_% of selected weeds under rye and spelt respect to the unweeded CNT was upheld by qualitative observation of AMF external hyphae development in presence of CC. The abundant extraradical mycelium on rye and spelt root cortex observed by SEM, together with its corresponding increase on weeds roots, particularly in *S. media* and *V. persica* in both the CC systems, confirmed their positive influence in potentially connecting adjacent roots through development of the external mycelium growth, also in some weed species usually not colonized by AMF to profitably distribute the soil resources (Teste et al., 2014).

Since the *M*% values for each plant species cannot describe how the plant–plant interactions affect the overall mycorrhizal colonization of diversified ecosystems, we used our data to calculate the MA%, as an indicator of the belowground functional biodiversity. Indeed, the agroecological service provided by agroecosystem mycorrhization depends on the ability of the mycorrhizal plants, such as the winter cereal CCs, to support the diversity and abundance of agronomically beneficial AMF taxa, optimize the ecosystem resources, and mediate positively the weed population dynamics through the AMF–CC–weed interaction (Vatovec, Jordan, & Huard, 2005).

The proposed MA% indicator would quantify the above‐described ecological service at the scale of the considered agroecosystems. In 2014, under less competitive environmental conditions, the CCs did not significantly affect the overall mycorrhizal colonization of the agroecosystem compared to the CNT. Conversely in 2015, due to increased mycorrhization of specific weeds, also offset by a corresponding increase of the CC contribution to AMF colonization, the MA% increased noticeably in both the cover cropped systems respect to the uncovered one.

To explain the sensitiveness of the system cover cropped with rye to environmental conditions, we can refer to its allelopathic properties. It was verified, in field and in vitro, that the presence of mycorrhizal mycelium on plant roots determined the increase of allelopathic compounds transfer, resulting in a reduced growth of target plants (Achatz et al., 2014). In our CC system, this preferential transport of allelochemicals through the AMF extraradical hyphae as belowground “highways” may be claimed as a strategy used by the rye to contain weeds (Barto et al., 2011). The extension of the rye bioactive zone, that corresponded to the extraradical hyphae development, can reduce the degradation of emitted allelochemicals by the soil microflora: In 2014, under high rainfall, this mechanism was evidently less effective, being influenced by the variability across the field (Kaur, Kaur, Kaur, Baldwin, & Inderjit., 2009). In 2015, the more competitive conditions led to the presence of only the rye and the *A. arvensis* in field: being AMF–host plants both the rye and *A. arvensis*, they boosted their root mycorrhizal colonization for overcoming competition with the other plants, as shown by the higher MA% under rye respect to the CNT (Torrecillas, Alguacil, & Roldán, 2012). This confirmed previous results on forage radish, which increased its mycorrhizal colonization in presence of rye (White & Wei, 2009).

In 2014, the MA% under spelt was mostly due to the CC and *S. media*, while in 2015 to the CC and *A. arvensis* contribution: the ability of spelt to boost the mycorrhization also in plants commonly recognized as non‐host species as the *S. media,* sharing the rhizosphere space and resources with such selected weeds, differed from that of rye, which instead addressed the mycorrhizal colonization of the agroecosystem mainly in favor of its own ecological dominance (Veiga, Howard, & Heijden, 2012; Veiga et al., 2011). In 2014 and 2015, although at different order of magnitude, the high MA% obtained under spelt confirmed its potential as agroecological service crop, more than the rye, able to support the AMF beneficial colonization, also under unfavorable climatic conditions.

Our results demonstrated the eco‐social role of root mycorrhizal colonization in mediating CC–weed interaction, although affected by the climatic conditions and the CC. Given the dependence of plant species density from its mycorrhization in field, the mycorrhization appears as an effective strategy played by the CC to contain weed, and by the weed to compete with CC.

The proposed MA indicator allows the quantification of the agroecological service supplied by the CC, in terms of increase or inhibition of the overall mycorrhization in a given agroecosystem, affecting weed emergence and development. It can indicate the agroecosystem resilience, resulting from plant–plant interference, allelopathic interactions and environmental and climatic conditions, so to be used as a potential indicator of the agroecological service provided by the CC.

Finally, our study assessed the extent of the linkage of specific crop traits to agroecosystem services, thus providing potential guidance to exploit suitable biodiversity‐based management options at field and farm scale, contributing to further develop the functional biodiversity theory (Bàrberi, 2015; Costanzo & Bàrberi, 2014).

## CONFLICT OF INTEREST

None declared.

## AUTHORS′ CONTRIBUTIONS

Alessandra Trinchera conceived the hypothesis of the research, the sampling of plant root apparatus, the proposed conceptual model and the new agroecological indicator, and the writing of the manuscript. Stefano Canali, Gabriele Campanelli, Corrado Ciaccia and Fabrizio Leteo designed the field trials. Gabriele Campanelli and Fabrizio Leteo managed the 2‐year experiment at CREA MOVELTE site in Monsampolo del Tronto (AP). Corrado Ciaccia recognized all weed species and collected density and biomass data of weed and CCs with Elena Testani. Elena Testani and Valentina Baratella evaluated plants root mycorrhization by optical microscopy. Alessandra Trinchera carried out SEM analysis on plant root. Valentina Baratella performed the statistical analysis. All authors contributed critically to the drafts. Stefano Canali revised the final draft and gave final approval for publication.

## Data Availability

The data will be archived in dryad (https://doi.org/10.1002/ece3.5125).
